# 中性粒细胞胞外诱捕网在肺癌中的研究进展

**DOI:** 10.3779/j.issn.1009-3419.2025.106.06

**Published:** 2025-03-20

**Authors:** Xu HAO, Yilin FENG, Anqi LU, Ying SUN, Jinchan XIA, Xue MEI, Long FENG, Min JIANG, Baiyan WANG, Huitong YANG

**Affiliations:** 450046 郑州，河南中医药大学医学院; Medical College of Henan University of Chinese Medicine, Zhengzhou 450046, China

**Keywords:** 肿瘤相关中性粒细胞, 中性粒细胞胞外诱捕网, 肺肿瘤, 肿瘤微环境, Tumor associated neutrophils, Neutrophil extracellular traps, Lung neoplasms, Tumor microenvironment

## Abstract

中性粒细胞胞外诱捕网（neutrophil extracellular traps, NETs）作为中性粒细胞活化后释放的网状复合结构，在恶性肿瘤病理进程中发挥关键调控作用。肺癌是全球范围内最常见的恶性肿瘤之一，发病率和死亡率均居高不下。近年研究发现，NETs通过其独特的病理机制参与肿瘤微环境动态调控，在肺癌演进过程中呈现出复杂的免疫调节特征，这一发现已逐渐成为肿瘤免疫学研究的热点领域。本文系统地梳理了NETs在肺癌领域的最新研究进展，深度剖析了NETs对肺癌发生发展的影响和在肺癌诊断中的潜在价值，以及靶向NETs防治肺癌的研究现状，旨在为提高肺癌患者治疗疗效和改善预后提供新的思路。

## Abstract (en)

Neutrophil extracellular traps (NETs), intricate reticular structures released by activated neutrophils, play a pivotal regulatory role in the pathogenesis of malignant tumors. Lung cancer is one of the most prevalent malignancies globally, with persistently high incidence and mortality rates. Recent studies have revealed that NETs dynamically modulate the tumor microenvironment through unique pathological mechanisms, exhibiting complex immunoregulatory characteristics during the progression of lung cancer, and this discovery has increasingly become a focal point in tumor immunology research. This paper provides a comprehensive review of the latest advancements in NETs research related to lung cancer, offering an in-depth analysis of their impact on lung cancer progression, their potential diagnostic value, and the current state of research on targeting NETs for lung cancer prevention and treatment. The aim is to propose novel strategies to enhance therapeutic outcomes and improve the prognosis for lung cancer patients.

肺癌是最常见的癌症类型之一，世界卫生组织国际癌症研究机构的最新数据^[1]^表明，2022年全球有近250万肺癌新发病例和超过180万例死亡病例。小细胞肺癌（small cell lung cancer, SCLC）和非小细胞肺癌（non-small cell lung cancer, NSCLC）分别占肺癌总数的15%和85%左右^[2]^。众所周知，肿瘤微环境（tumor microenvironment, TME）的免疫抑制特性与肺癌的发生发展密切相关^[3]^。而中性粒细胞是TME中的关键免疫细胞，可通过吞噬作用、脱颗粒以及生成中性粒细胞胞外诱捕网（neutrophil extracellular traps, NETs）参与调节TME^[4]^。2013年Berger-Achituv等^[5]^首次提出尤文氏肉瘤中的肿瘤相关中性粒细胞（tumor-associated neutrophils, TANs）活化后产生NETs，开启了NETs在肿瘤领域中的研究。

目前，有关NETs生成的作用机制尚未被完全阐明。研究^[6,7]^认为，NETs是中性粒细胞在受到自身或外界刺激后形成的胞外DNA网状结构，由DNA骨架和多种蛋白质组成，主要包括中性粒细胞弹性蛋白酶（neutrophil elastase, NE）、髓过氧化物酶（myeloperoxidase, MPO）、基质金属蛋白酶9（matrix metalloproteinase 9, MMP9）、肽酰基精氨酸脱亚胺酶4（peptidyl arginine deiminase 4, PAD4）以及瓜氨酸组蛋白H3（citrullinated histone H3, Cit-H3）等。中性粒细胞形成NETs的途径名为NETosis，主要分为三类：溶解型NETosis、非溶解型NETosis和线粒体NETosis^[7]^（图1）。目前研究最多的NETs形成途径是溶解型NETosis，即在NETs形成过程中伴随氧化应激驱动的中性粒细胞死亡，是不同于细胞凋亡和细胞坏死的新型细胞死亡方式^[8]^。中性粒细胞在受到佛波醇-12-肉豆蔻酸酯（phorbol 12-myristate 13-acetate, PMA）、免疫复合物、自身抗体或晶体等刺激后，激活蛋白激酶C（protein kinase C, PKC）和丝氨酸/苏氨酸激酶（serine/threonine kinase, Raf）/丝裂原活化蛋白激酶激酶（mitogen-activated protein kinase kinase, MEK）/细胞外调节蛋白激酶（extracellular regulated protein kinases, ERK）信号通路，以钙离子依赖性的方式诱导NADPH氧化酶（NADPH oxidases, NOX）活化并释放活性氧（reactive oxygen species, ROS），致使颗粒膜和细胞核膜崩解^[9]^。在该过程中焦孔素D（gasdermin D, GSDMD）可协助NETs排出，这种溶解性细胞死亡过程通常需要2-5 h^[7,10]^。中性粒细胞表面Toll样受体（Toll-like receptor, TLR）经金黄色葡萄球菌、革兰氏阴性细菌、真菌、病毒、脂多糖（lipopolysaccharides, LPS）或血小板等刺激时，PAD4及下游信号通路被激活，促进NETs形成并以囊泡的形式排出，此为非溶解型NETosis。在该途径中细胞内钙浓度增加是主要因素，NOX的激活和ROS的生成并非必要条件^[11⇓-13]^。此时，中性粒细胞仍存活并保留其功能，该过程仅需5-60 min^[7]^。值得注意的是，有研究^[14]^描述了一种新的NETs生成途径，即线粒体DNA（mitochondrial DNA, mtDNA）参与的NETosis。中性粒细胞受粒细胞巨噬细胞集落刺激因子（granulocyte/macrophage colony-stimulating factor, GM-CSF）的作用，进而用LPS或补体因子C5a（complement component 5a, C5a）刺激后，可释放大量mtDNA而不含核DNA，以ROS依赖的方式诱导NETs生成。这个过程并不伴随中性粒细胞的死亡，因此认为它可能与非溶解型NETosis类似^[14]^。肿瘤细胞分泌的白细胞介素-8（interleukin-8, IL-8）可能是线粒体NETs的主要诱导剂^[15]^，同时自噬也参与调控线粒体NETosis的发生^[16]^。

**图1:**
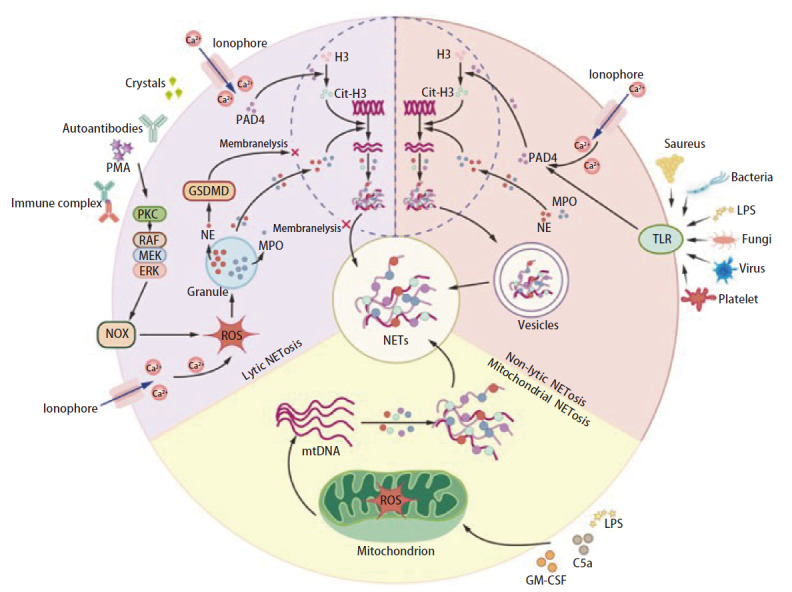
中性粒细胞胞外诱捕网的生成机制

值得注意的是，溶解型NETosis和非溶解型NETosis的诱发因素似乎在某种程度上重叠，然而目前尚不清楚为何某一特定因素能够启动特定途径，而不是其他途径^[17,18]^。此外，自噬调控线粒体NETosis的发生，可能与自噬的程度和诱导自噬的特定方式有关。在某些情况下自噬还能够抑制NETs的过度释放^[19]^。虽然所涉及的机制尚未被完全阐明，但自噬诱发NETs可能是把双刃剑：一方面，自噬通过触发NETs形成防止病原体感染^[20]^；另一方面，自噬驱动的NETosis也可能损害肺组织，导致细胞死亡和恶性转化^[21]^。

就功能而言，NETs具有双重作用，不仅可促进肿瘤增殖和远处转移，还可诱导肿瘤细胞凋亡，发挥抗肿瘤作用^[22,23]^。本文系统地梳理了NETs在肺癌中的作用和NETs在肺癌诊断方面的价值，以及靶向NETs防治肺癌的研究现状，旨在为开发新型肺癌治疗手段提供新的思路。

## 1 NETs对肺癌的影响

临床研究^[24]^显示肺癌患者体内NETs呈现出高表达状态，同时动物实验^[25]^表明，Lewis肺癌小鼠肿瘤组织中NETs相关蛋白MPO和Cit-H3的表达量增多。上述现象说明NETs对肺癌的发生发展主要起到促进作用，但也有研究^[26]^表明，NETs可调控TME并发挥促肿瘤和抗肿瘤的双重效应。

### 1.1 NETs的促肿瘤作用

#### 1.1.1 NETs促进肿瘤细胞的增殖、侵袭、迁移和血管生成

肿瘤细胞的增殖、侵袭、迁移和血管生长均是癌症进展的重要标志。在NETs增强肿瘤细胞增殖方面的研究^[27]^中发现，敲除Lewis肺癌小鼠NETs相关蛋白PAD4，肺肿瘤的生长速度减缓。此外，NETs释放NE可以刺激肿瘤细胞TLR4/p38/PGC1信号转导，增强肿瘤线粒体功能和生物发生，驱动小鼠结直肠癌的生长^[28]^。Han等^[29]^在肺癌细胞系A549和PC9中观察到沉默磷脂爬行酶1（phospholipid scramblase 1, PLSCR1）后，NETs显著减少并伴有肿瘤细胞的增殖减弱。除了直接作用外，NETs还可参与调控其他免疫细胞的功能，如NE（NETs的主要成分）能够诱导巨噬细胞向促肿瘤表型极化，加速肺癌细胞A549和H1299的增殖^[30]^。此外，肿瘤细胞分泌的细胞外RNA（extracellular RNA, exRNA）作为细胞间通讯工具被包裹在囊泡中，可参与诱导NETs生成，进一步加速肿瘤细胞增殖。如Lewis肺癌细胞释放的exRNA能够降低小鼠肺泡上皮细胞MLE-12中IL-1β的分泌量，诱导中性粒细胞发生NETosis。释放的NETs可以下调MLE-12细胞中紧密连接闭合蛋白5的水平，引起上皮细胞坏死，通过正反馈途径加速exRNA释放，促进肺癌的进展^[31]^。但有关exRNA在TME中诱导NETs生成的具体作用机制尚需进一步明确。

计算机模拟结果提示，在NETs的影响下肺癌细胞侵袭和迁移能力显著增强^[32]^。研究^[33]^表明在循环系统中NETs可以捕获播散性肿瘤细胞以支持转移性扩散，NETs能够为肿瘤细胞在远处结合提供富含DNA和纤连蛋白的环境。这些部位长时间暴露于NETs会激活Notch 1信号转导并触发上皮间充质转化（epithelial-mesenchymal transition, EMT），诱导肿瘤细胞发生侵袭和迁移。另外的观点认为，NETs通过与TME中其他免疫细胞发生串扰介导肿瘤细胞的侵袭和迁移。如肺癌患者中性粒细胞释放的NETs，可刺激巨噬细胞分泌IL-1β、IL-6、IL-18和肿瘤坏死因子α（tumor necrosis factor-alpha, TNF-α）等促炎因子，增强与巨噬细胞共培养体系中A549肺癌细胞的侵袭和迁移能力^[34]^。此外，长链非编码RNA（long non-coding RNA, lncRNA）参与抑制肿瘤细胞的侵袭和迁移，而NETs可调控其表达。如NETs可以下调lncRNA MIR503HG并激活核因子-κB（nuclear factor kappa-B, NF-κB）/NLRP3，促进IL-18和IL-1β的释放，增强肿瘤细胞A549和SK-MES-1的侵袭和迁移能力^[24]^。NETs及其相关内容物在促血管生成方面也起着至关重要的作用。MPO以TLR4依赖的方式诱导内皮细胞的成管效应^[35,36]^。此外，NETs中的可溶性组织蛋白酶G能够切割pro-MMP9介导转化生长因子-β（transforming growth factor-β, TGF-β）活化，刺激乳腺肿瘤诱导溶骨病灶血管的生成^[37]^。

#### 1.1.2 NETs诱导免疫抑制微环境的形成

T淋巴细胞免疫功能的抑制与癌症进展有关^[38]^。NETs能够通过多种方式调节免疫细胞功能，建立免疫抑制生态位。一方面，NETs的DNA骨架成分（NETs-DNA）可充当物理屏障，保护肿瘤细胞免受自然杀伤（natural killer, NK）细胞和细胞毒性T淋巴细胞（cytotoxic T lymphocytes, CTLs）等细胞毒性细胞的杀伤。同样地，体内研究^[39]^发现，NETs还可以阻挡Lewis肺癌小鼠皮下移植瘤中细胞毒性细胞与肿瘤细胞的接触。这种作用在体外共培养模型中也被发现，中性粒细胞释放的NETs能够包绕肿瘤细胞，削弱CTLs或NK细胞等细胞毒性免疫细胞对肿瘤细胞的杀伤作用^[39]^。另一方面，NETs促进T细胞耗竭，保护肿瘤细胞逃避免疫细胞的杀伤作用，如NSCLC患者肿瘤组织中NETs的表达与具有细胞毒性的CD8^+ ^T细胞浸润呈负相关^[40]^。在进一步的功能研究^[41]^中发现，与NETs共培养后，包括淋巴细胞活化基因3（lymphocyte-activation gene 3, LAG3）和T淋巴细胞免疫球蛋白黏蛋白3（T-cell immunoglobulin and mucin domain-containing protein 3, TIM3）等的CD8^+^ T细胞耗竭基因表达升高，使得T细胞功能发生障碍，推动NSCLC进展。同样地，NETs可削弱肝癌小鼠T细胞的抗肿瘤免疫效应。其中，NETs-DNA能够与CD8^+ ^T细胞表面跨膜和螺旋-线圈结构域6（transmembrane and coiled-coil domains 6, TMCO6）的N端结合，干扰T细胞受体通路激活和NF-κB/p65核易位，抑制T细胞活化和增殖，下调细胞毒性相关介质如颗粒酶B和干扰素-γ的水平^[42]^。有趣的是，NETs还可能通过代谢重编程诱导调节性T细胞（regulatory T, Treg）分化。在有关小鼠非酒精性脂肪性肝炎的研究^[43]^中发现，NETs能够激活TLR4信号传导，增加幼稚CD4^+^ T细胞的氧化磷酸化并诱导分化为Treg，加速肝炎向肝细胞癌进展。然而，这种作用是否在肺癌中存在仍需验证。

#### 1.1.3 NETs推动肿瘤的远处转移

目前认为，包括肺癌在内多数癌症的远处转移是患者预后不良的标志。近期研究^[44⇓-46]^发现，NETs可参与肿瘤的远处转移。循环肿瘤细胞（circulating tumor cells, CTCs）是癌症转移的前体，在预测远处转移、评估预后和监测治疗反应等方面具有重要的临床价值^[47]^。一方面，NETs可捕获CTCs，协助CTCs播散并促进CTCs与靶器官的黏附^[44]^。循环NETs通过捕获扩散的肿瘤细胞，引导其以跨血管屏障的转运机制发生外渗，建立增殖性转移灶。血流经过肝窦时，NETs表达的β1整合素可与A549肺癌细胞表面受体结合，促进A549肺癌小鼠肝脏微转移^[45]^。同样地，败血症诱发的NETosis会促进小鼠Lewis肺癌肝转移^[46]^。PAD4缺陷的Lewis肺癌小鼠，肝脏和肺转移率降低，这进一步支持了NETs在建立肿瘤转移微环境中的作用^[25]^。另一方面，NETs可以通过增强CTCs的存活、侵袭和外渗来促进肿瘤转移。例如，NETs可以通过形成物理屏障和分泌免疫抑制细胞因子来保护CTCs免受免疫毒性细胞（如NK细胞）介导的细胞毒性^[46]^。NETs还可以通过释放蛋白酶或ROS降解细胞外基质（extracellular matrix, ECM），增加血管通透性，促进CTCs外渗^[48]^。NETs促进肿瘤远处转移的一种机制为NETs-DNA可作为一种趋化因子，通过受体-配体相互作用吸引肿瘤细胞。研究^[49]^发现，肿瘤表面的卷曲螺旋结构域含蛋白25（coiled-coil domain containing 25, CCDC25）是NETs-DNA的配体，它与NETs-DNA结合能够激活并触发ILK/β-Parvin/RAC1/CDC42信号转导，增强乳腺癌细胞MDA-MB-231的运动能力，促进肿瘤微转移灶的形成。此外，活化的血小板经TLR4-ERK5轴促进手术应激条件下NETs对CTCs的捕获并加速结肠癌向肺癌的远处转移^[47]^。第二种作用机制认为，NETs-DNA能够诱导EMT以增加肿瘤细胞的侵袭性。如使用NETs孵育肿瘤细胞，可以下调E-钙黏蛋白并上调间充质蛋白（包括纤连蛋白、N-钙黏蛋白和波形蛋白）的表达，赋予肿瘤细胞离开原发肺癌部位的能力。在进一步的机制探究^[24]^中发现，NETs通过下调lncRNA MIR503HG，激活NF-κB/NLRP3信号转导，增加肺癌转移裸鼠肺内的转移性结节。此外，学者们认为转移性肿瘤细胞可在远处组织中长时间进入休眠状态，而NETs可唤醒转移后休眠肿瘤细胞，研究^[50]^发现，NETs中的NE和MMP9可降解层黏连蛋白111等EMT相关蛋白，激活α3β1整合素信号转导，刺激乳腺癌休眠模型小鼠的播散性肿瘤细胞结束休眠并发生增殖和肺转移。总的来说，NETs在引导肿瘤细胞到达转移生态位并唤醒转移后休眠肿瘤细胞方面发挥着至关重要的作用。

#### 1.1.4 NETs阻碍抗肿瘤治疗

目前认为NETs是癌症患者不良预后的重要影响因素，参与诱导肿瘤对放化疗和免疫治疗的耐药性，严重阻碍抗癌疗法的有效性。EMT与肺癌化疗耐药性相关，而正如上文所述NETs参与诱导NSCLC的EMT^[24]^；同时，NETs刺激乳腺癌细胞中TGF-β活化，促进肿瘤EMT并介导化疗耐药^[51]^。目前，研究人员对中性粒细胞在放射治疗反应中的作用理解尚不完整。有研究者^[52]^认为，中性粒细胞会抵消放射治疗的疗效，如高表达葡萄糖转运蛋白1（glucose transporter type 1, GLUT1）的中性粒细胞的葡萄糖代谢提高，将限制肺腺癌小鼠对放疗的敏感性。在肺癌中这一作用是否有NETs参与尚未加以证实，而在对放射治疗无反应或低反应的膀胱癌患者体内观察到大量NETs。经放射治疗后浸润性膀胱癌患者的中性粒细胞在肿瘤组织中聚集并产生NETs，介导对放射治疗的抗性。在进一步的机制研究^[53]^中发现，放疗诱导癌细胞释放HMGB1，触发TLR4信号传导并增加NETs的释放；反之，使用脱氧核糖核酸酶I（deoxyribonuclease I, DNase I）或中性粒细胞弹性蛋白酶抑制剂部分消除NETs，可改善患者对放射治疗的总体反应性。

癌症免疫疗法旨在增强免疫系统识别和攻击肿瘤细胞的能力。然而，相当多的患者对免疫疗法无反应或产生耐药性，而NETs参与这种作用的发生。在有关NSCLC的临床研究^[54]^中发现，中性粒细胞释放的NETs会降低免疫检查点抑制剂（immune checkpoint inhibitors, ICIs）的疗效；同时，ICIs治疗前后的血浆NETs水平与NSCLC患者的不良总生存期和无进展生存期有关。一种观点认为，免疫调节程序性死亡配体1（programmed cell death ligand 1, PD-L1） 在NETs中表达，提示NETs本身可发挥一定的免疫抑制功能。NSCLC患者使用控制轻度疼痛的非处方药对乙酰氨基酚（Acetaminophen, APAP）后，抗细胞凋亡程序性受体1（programmed cell death-1, PD-1）的疗效显著降低。这种作用同样在Lewis肺癌小鼠中被观察到，作用机制可能在于APAP诱导中性粒细胞活化并加速NETs的生成^[55]^。另一种观点则认为，NETs能够调节其他免疫细胞的功能，促进对ICIs有抵抗力的免疫抑制微环境的形成。正如前文所述，NETs的DNA结构可充当物理屏障，阻挡肿瘤细胞与细胞毒性免疫细胞之间的接触。当NETs存在时可削弱来自于肺癌小鼠的NK细胞和CD8^+ ^T细胞对Lewis肺癌细胞的细胞毒作用。反之，腹膜内注射GSK484对NETosis进行药理学抑制，肿瘤对PD-1和细胞毒性T淋巴细胞相关蛋白4（cytotoxic T lymphocyte-associated antigen-4, CTLA-4）免疫双检查点阻断的敏感性增加^[39]^。因此，与靶向NETs联合可能是提高ICIs抗肿瘤作用的可行途径。

### 1.2 NETs的抗肿瘤作用

NETs除上述促肿瘤作用外，还可通过诱导肿瘤细胞凋亡和增强T细胞免疫发挥抗肿瘤作用，如褪黑素处理的CD11b^+ ^Ly6G^+^中性粒细胞高表达诱导型一氧化氮合酶（inducible nitric oxide synthase, iNOS）和NOX2，同时NETs相关蛋白MPO和Cit-H3也显著上调，诱导胰腺癌小鼠肿瘤细胞凋亡。使用ROS清除剂N-乙酰半胱氨酸和PAD4抑制剂GSK484共同干预中性粒细胞，褪黑素介导的肿瘤抑制作用减弱，初步提示褪黑素介导的肿瘤细胞凋亡与NETs有关^[56]^。另外一项研究也证实了NETs参与诱导肿瘤细胞的凋亡，Cao等^[57]^将肿瘤坏死因子相关凋亡诱导配体基因敲入中性粒细胞并刺激生成NETs，与人结肠癌细胞COLO-205共培养可诱导肿瘤细胞凋亡。在进一步的探究^[58]^中发现，作为NETs的重要组成成分，NE除了通过促进转移和重塑TME来调节肿瘤生长外，还具有特异性的抗肿瘤作用，具体表现在，NE的蛋白水解活性会切割释放CD95的死亡结构域，导致癌细胞死亡而不影响正常免疫细胞；同时，NE处理荷瘤小鼠可增加CD8^+^ T细胞浸润，抑制肿瘤生长和远处转移。此外，NETs还可以增强T细胞的杀伤作用。Tillack等^[59]^发现NETs上调ZAP70磷酸化水平，促进人CD4^+ ^T淋巴细胞表达活化相关蛋白CD69、CD25，从而降低CD4^+ ^T细胞活化阈值。这种作用在体内也被证实，膀胱癌移植瘤小鼠皮下注射NETs-DNA和NETs，可观察到大量T淋巴细胞募集，且伴有肿瘤体积的明显缩小。此外，据报道用于治疗膀胱癌的卡介苗（bacillus calmette-guérin, BCG）也能够诱导NETs生成，并促进T细胞、单核细胞和巨噬细胞募集以控制肿瘤生长^[60]^。上述研究均提示NETs可能发挥抗肿瘤作用，但目前关于NETs在肺癌进展中发挥抗肿瘤活性的研究尚未见报道。

综上所述，在不同的TME中，NETs的作用存在差异。值得注意的是，NETs在正常组织和肿瘤组织中的生成量并不均匀，且不同位置形成的NETs具有不同的功能。例如在血管内、癌旁组织、休眠与增殖癌细胞附近、不同类型的ECM或免疫细胞浸润区域形成的NETs可能会产生不同的影响^[59⇓⇓-62]^。因此，进一步了解NETs生成的空间定位对于揭示NETs与肿瘤的关系至关重要。

## 2 NETs在肺癌诊疗方面的潜在价值

中性粒细胞减少会引发严重感染，限制了靶向中性粒细胞的临床应用。然而，鉴于NETs在癌症发生和进展中起着的关键作用，靶向NETs可能是一种潜在的抗肿瘤策略。同时，在血液循环或肿瘤组织中检测到NETs及其相关分子。因此，它们越来越多地被视为肿瘤诊断和预后的生物标志物。

### 2.1 诊断

近年来NETs相关分子被认为是癌症的潜在生物标志物，例如Cit-H3、NE和MPO增加已被证实存在于原发性肿瘤和转移性病变中。在临床诊疗中发现，肺癌患者体内中性粒细胞大量浸润，NETs增多^[63]^。此外，循环IL-8水平、中性粒细胞与淋巴细胞比值（neutrophil-to-lymphocyte ratio, NLR）和NETs水平与NSCLC患者免疫治疗疗效呈负相关^[64]^。同样地，血清Cit-H3、IL-8和C反应蛋白被联合用于预测NSCLC患者对ICIs的敏感性^[65]^。血清NETs含量、CD8^+^ T细胞和肿瘤细胞阳性比例评分的组合模型已被用于预测PD-1抑制剂对NSCLC患者的治疗疗效^[66]^。此外，NETs还可以作为肺腺癌患者临床预后和化疗治疗耐药性的指标^[67]^。因此，目前认为NETs可作为临床诊断和治疗肺癌的高敏感型和特异性生物标志物。为进一步提高检测的灵敏性，研究人员构建了串联锁定荧光NETosis报告系统^[68]^，在这个检测系统中NETosis Reporter 1（TNR1）仅在NE和蛋白酶G同时存在的情况下才会激活荧光信号，用于评估癌症免疫治疗的预后情况，实现了无创监测NETosis。虽然NE和MPO是NETs的两个重要组成部分，但它们在中性粒细胞活化初期即产生，并非在NETs形成过程中产生，这提示今后仍需要探索更为精确的检测标志物。

鉴于此，研究人员发现了另一种非侵入性、经济高效的检测手段，即以循环游离DNA（circulating free DNA, cfDNA）作为检测指标，确定释放至血液中NETs的水平。由组蛋白和双链DNA组成的核小体，属于cfDNA的一种。研究^[69]^表明，第二轮和第三轮化疗前，低水平循环核小体可增强NSCLC患者治疗疗效。此外，尽管cfDNA可部分反映肿瘤的发生，但它并不是可靠的NETs指标。因为除了癌细胞外，由其他疾病引发的细胞凋亡或坏死也可能导致血浆中cfDNA水平的升高。令人惊喜的是，研究发现血清Cit-H3的水平以及循环MPO-DNA或NE-DNA复合物与多种癌症的诊断和/或进展有关，且特异性更高。这些标志物能够将NETs与其他类型的非肿瘤衍生cfDNA区分开来，从而增加了靶向治疗的可能性。当前，Cit-H3或NE已在临床试验中用作NETs生物标志和预后指标，以评估包括胰腺癌、肺癌、乳腺癌和结直肠癌在内多种癌症患者血栓形成的风险^[70]^。综上表明，抑制NETs生成不仅可增强机体抗肿瘤能力，还能够提高肿瘤免疫治疗的疗效，从而改善癌症患者的预后。然而，NETosis发生在多种实体瘤，分布于肿瘤组织和循环中的NETs个体差异性较大；同时，评估组织中肿瘤相关NETs的检测仍处于起步阶段，尚需深入探究。

### 2.2 治疗

癌症与NETosis存在关联这一发现为NETs作为肿瘤治疗靶点提供了理论基础，故药理学靶向NETs将发挥巨大的潜在价值。目前，阻断NETs的手段主要集中于以下两个方面，阻止NETs的形成及破坏NETs的结构或消除癌细胞与NETs之间的相互作用（表1）。虽然当前针对NETs生成及促进其降解的药物较为有限，但NETs形成级联反应关键酶的部分抑制剂已进入临床测试阶段。

**表1:** 肺癌中针对NETs的抑制剂和药物

Major impact	Targets	Inhibitors	Type of cancer	Reference
Promote degradation of NETs	DNA	DNase I	Non-small cell lung cancer	^[24,25]^
Inhibiting NETs formation	NE	NEi	Non-small cell lung cancer	^[25]^
Inhibiting NETs formation	NE	Sivelestat	Non-small cell lung cancer	^[25]^
Inhibiting NETs formation	NE	NE anti-body	Non-small cell lung cancer	^[34]^
Blocking interaction with tumor cells	IL-β1	4B4	Non-small cell lung cancer	^[45]^
Inhibiting NETs formation	NE	GW311616A	Non-small cell lung cancer	^[46]^
Blocking the pathway	CXCR1/CXCR2	Navarixin (NCT03473925)	Non-small cell lung cancer	^[71]^
Inhibiting NETs formation	Oxidative stress	Liraglutide	Non-small cell lung cancer	^[72]^
Inhibiting NETs formation	Oxidative stress	Methyl syringate	/	^[73]^
Inhibiting NETs formation	Oxidative stress	Resveratrol	/	^[74]^
Inhibiting NETs formation	MPO/Cit-H3	Yi Qi Chu Tan Formula	Non-small cell lung cancer	^[75]^
Inhibiting NETs formation	PAD4	CI-amidine	Non-small cell lung cancer	^[76]^
Inhibiting NETs formation	PAD4	Didang decoction	Non-small cell lung cancer	^[76]^
Blocking the pathway	IL-8	Didang decoction	Non-small cell lung cancer	^[76]^
Blocking the pathway	IL-8	DNase I	Non-small cell lung cancer	^[76]^
Inhibiting NETs formation	NE	Curcumin	Non-small cell lung cancer	^[77]^

DNase I: deoxyribonuclease I; NEi: neutrophil elastase inhibitor; NE: neutrophil elastase; MPO: myeloperoxidase; Cit-H3: citrullinated histone H3; PAD4: peptidyl arginine deiminase 4; IL-β1: interleukin-β1; CXCR1: C-X-C motif chemokine receptor 3.

#### 2.2.1 抑制NETs生成

PAD4是NETs的主要成分之一，可促进组蛋白瓜氨酸化增强NETosis。CI-amidine是一种不可逆的泛PAD抑制剂，对PAD家族的同工酶均有效。GSK-484是一种可逆的选择性PAD4抑制剂，在阻断NETs形成和肿瘤进展方面表现出与DNase I类似的效果^[78]^。Li等^[79]^在骨髓瘤小鼠中尝试了一种新型PAD4特异性抑制剂BMS-P5，发现与其他非选择性PAD抑制剂CI-amidine和GSK-484相比，BMS-P5对NETosis的抑制作用更为显著。同样地，PAD4抑制剂YW3-56和5i可下调中性粒细胞和4T1乳腺癌细胞Cit-H3的水平，抑制NETs生成，阻止小鼠体内肿瘤的生长和转移^[80]^。另外，在临床前结肠癌肝转移小鼠中，泛PAD2/PAD4抑制剂YW4-03可缓解手术应激引发的NETs介导的炎症风暴^[70]^。尽管抑制PAD4在实验环境中非常成功，但目前尚不存在针对PAD4的药物被批准用于临床。

除了直接抑制NETs生成外，靶向NETs生成的上游介质也能达到类似的效果。如肿瘤细胞分泌的IL-8（也称为CXCL8）是中性粒细胞的另一种关键激活剂和化学引诱剂，参与诱导NETosis发生^[81]^。作为下游响应，CXCR1/CXCR2对IL-8反应强烈。因此，阻断IL-8通路及其受体CXCR1/CXCR2将成为一种有前途的抗肿瘤策略。目前，CXCR1/CXCR2抑制剂正在进行临床试验，例如Navarixin（NCT03473925）、SX-682（NCT05604560）、Reparixin（NCT02370238）和AZD5069（NCT03177187）。其中，Navarixin是人类CXCR1/CXCR2的强效选择性拮抗剂之一，临床试验正在评估它与帕博利珠单抗联合治疗NSCLC、去势抵抗性前列腺癌或微卫星稳定性结直肠癌的效果^[71]^。在有关作用机制方面的研究^[82]^中发现，敲除CD276可下调CXCL1，阻碍CXCL1/CXCR2轴介导的肿瘤与中性粒细胞间的相互作用，减少NETs生成，抑制小鼠食管鳞状细胞癌的生长。此外，也有报道^[83]^称肝细胞分泌的假丝氨酸蛋白酶PRSS35能够切割串联赖氨酸识别基序并促进CXCL2降解，阻碍中性粒细胞的募集和NETs生成，从而减缓肝癌小鼠体内肿瘤生长。

除了直接参与NETs形成的关键分子外，氧化应激和NETs生成相关信号转导亦是影响NETosis发生的重要因素。在ROS影响NETs生成的研究^[72]^中发现，用于II型糖尿病治疗的药物利拉鲁肽可抑制机体氧化应激，下调Lewis荷瘤小鼠循环NETs的水平，协同提高抗PD-1的疗效。同样地，麦卢卡蜂蜜的活性成分丁香酸甲酯能够有效抑制人外周血原代中性粒细胞ROS活性和NETs形成^[73]^。除了小分子药物外，富含组氨酸的糖蛋白能够结合中性粒细胞表面的Fc受体，抑制NF-κB/MAPK/p38信号转导和ROS生成，阻碍NETosis发生，降低肝癌肺转移率^[84]^。此外，具有调控氧化应激反应的中药也会影响中性粒细胞NETosis发生。在肺癌研究^[74]^中，白藜芦醇表现出良好的抗肿瘤作用，通过下调中性粒细胞过氧化氢的水平，阻碍染色质的解聚以及PMA、LPS诱导的NETs释放。淫羊藿素因其具有调节免疫功能及抗肿瘤等多种生物活性在肺癌治疗中受到关注，可抑制MEK/ERK/p38或PI3K/AKT信号转导，减少ROS生成，下调PAD2和MPO水平，抑制NETs生成^[85]^。在调控NETs生成相关信号转导方面，中药单体、中药复方和中成药注射液也发挥着关键作用。传统中药朱砂与氧化汞制备的含汞制剂可以阻止糖尿病小鼠中性粒细胞ERK1/2的激活，下调NETs相关蛋白MPO、PAD4和Cit-H3的水平^[86]^。进一步的研究^[75]^发现，益气除痰方作为治疗肺癌的有效方剂，可下调肿瘤组织中NETs相关蛋白Cit-H3、MPO及其EMT相关蛋白MMP9和细胞间黏附分子-1（intercellular cell adhesion molecule-1, ICAM-1）的水平，从而达到抑制Lewis小鼠肿瘤生长的作用。此外，抵当汤作为理血剂，具有破血通经、逐瘀消癥的功效。作为临床常用药物，抵当汤在恶性肿瘤和糖尿病等疾病中发挥显著疗效。Zeng等^[76]^发现抵当汤可抑制Lewis小鼠肿瘤体积的增长和相关血栓的形成，这个作用可能是通过调控NF-κB/IL-8从而阻止NETs积累实现的；同时，抵当汤也可下调NETs相关蛋白PAD4的水平，抑制小鼠原代中性粒细胞NETs的形成。在中药注射液调控NETs生成方面的研究中发现，具有化瘀解毒作用的血必净注射液常被用于临床肺癌化疗后肺部感染、重症肺炎、肺损伤以及脓毒血症的救治，研究^[87]^表明，血必净注射液也能够调控NETs生成，即阻断Raf/MEK/ERK激活，减少NETs释放。同样地，热毒宁注射液发挥清热解毒、祛风除湿的作用，能够显著改善原发性支气管肺癌患者的中医证候，有效控制感染。在一项体内实验研究^[88]^中发现，热毒宁注射液下调NETs相关蛋白PAD4及ERK1/2的磷酸化水平，改善LPS诱导的小鼠急性肺损伤。然而，目前尚缺乏中药调控NETs治疗肺癌相关NETs有关的临床研究。

#### 2.2.2 促进NETs降解

使用药物破坏NETs的结构，或消除癌细胞与NETs之间的相互作用也将成为阻断NETs的手段。正如前文所述，NETs是一种胞外DNA网状结构，由DNA骨架和多种蛋白质组成。而DNase可用于特异性降解DNA，实现靶向DNA骨架降解NETs的目的。目前，DNase I已被美国食品药品监督管理局批准用于囊性纤维化的治疗，同时在包括肺癌在内的多种肿瘤疾病的临床前模型中显示出治疗价值，它能够破坏NETs诱导的肿瘤转移性微环境^[25,45,46]^。在深入研究^[24]^中发现，DNase I处理不仅可抑制NETs诱导的肺癌细胞A549和SK-MES-1的侵袭和迁移，还可以减轻EMT，阻止肺癌转移。另有研究^[75]^揭示，DNase I对NETs的靶向作用可能是通过下调肺癌小鼠IL-8水平实现。此外，作为一种重组人DNase，Pulmozume已在III-IV期头颈癌患者中开展随机、安慰剂对照试验，以评估其安全性、耐受性和治疗效果。然而，DNase的全身生物分布可能会引起一定的安全问题，如损害宿主对感染的免疫防御等。鉴于此，Chen等^[89]^设计了一种纳米递送平台，通过近红外光照射将DNase特异性递送至肿瘤原发部位以及转移定植部位，同时增加T淋巴细胞和NK细胞等细胞毒性免疫细胞与小鼠肿瘤细胞的接触，提高癌症免疫治疗的敏感性。值得注意的是，由于NETs阻断剂可以提高包括化疗在内的常规疗法的疗效，研究人员^[90]^构建了以化疗药物紫杉醇为核心、内含DNase I的纳米制剂，经检测发现这种纳米制剂可有效抑制A549肺癌裸鼠的肿瘤增殖和远处转移。除DNA骨架外，多种蛋白质参与构成NETs，其中，NE在启动染色质去浓缩、促进NETosis发生过程中发挥重要作用。使用抗NE抗体降解NETs，可部分抵消NETs对肺癌细胞A549迁移和侵袭能力的促进作用^[34]^。在临床前肺和胃肠道肿瘤模型中使用Sivelestat对NE进行药理学抑制可防止肝和肺转移^[25]^。此外，GW311616A是一种长效选择性NE抑制剂，它的口服半衰期长、亲和力高。在全身性脓毒血症小鼠中，GW311616A全身给药可有效干扰NE活性，抑制NETs诱导的H59肺癌细胞肝转移^[46]^。值得注意的是，与GW311616A相比，DNase I和PAD4抑制剂均面临循环半衰期短的问题，这限制了全身给药的疗效。有趣的是，中药及中药化合物单体也可以降解NE以阻碍肿瘤进展。例如，姜黄素是姜黄的主要活性成分，具有抗肿瘤、抗炎、抗氧化等多种药理活性，用于NSCLC、胃癌、结直肠癌等多种实体肿瘤的治疗且效果明显^[77,91,92]^。在有关调控中性粒细胞NETosis的研究^[77]^发现，姜黄素能够调控PI3K/AKT，对抗NE诱导的肺癌细胞A549增殖。

有学者提出，消除肿瘤细胞与NETs之间的相互作用，可能是一种有效的抗肿瘤策略。正如前文所述，肿瘤细胞跨膜蛋白CCDC25可以充当细胞外DNA受体，它通过结合NET-DNA协助乳腺癌细胞发生肝转移，故认为CCDC25抗体将成为干扰CCDC25-NETs复合物形成以阻断肿瘤转移的可靠手段^[49]^。此外，整合素在肿瘤细胞和中性粒细胞上的表达，被认为是促进肿瘤和NETs相互作用的关键调节因子。将缺乏β1整合素的中性粒细胞过继至耗尽中性粒细胞的小鼠脾内，可观察到肺癌细胞的肝窦黏附作用显著降低^[45]^。其他影响肿瘤细胞与NETs结合的因素也可能成为今后抗肿瘤治疗的潜在靶点，尚需进一步探索。

鉴于NETs是放疗、化疗和ICIs耐药性的重要影响因子，因此未来临床试验有必要对NETs阻断剂与其他抗肿瘤策略加以联合来提高治疗效果。

## 3 总结和展望

越来越多的临床和临床前研究为肿瘤细胞本身或其周围环境引起的NETosis提供了更为直观深入的证据。反之，NETs的形成还可以进一步支持肿瘤发生和发展。后续的机制研究揭示了TME中NETs、肿瘤细胞和免疫细胞之间错综复杂的相互作用，将为更多基于NETosis的新型抗肿瘤疗法进入临床提供可能。鉴于对NETs在免疫细胞功能重塑和免疫抑制微环境形成方面理解的不断加深，针对NETosis的疗法不仅可以抑制转移性定植，还可以恢复机体的肿瘤免疫监视功能。但NETs具有促肿瘤和抗肿瘤的双重作用，给靶向NETs的研究增加了挑战性。本文不仅总结NETs的形成机制，还进一步阐述了NETs在肺癌发生发展、诊断和防治中的作用，但目前有关NETs的研究尚存在一些局限性。首先，不同表型的TANs释放的NETs是否存在差异；不同肿瘤类型和肿瘤分期中的NETs功能是否不同，以及其作用机制还需深入研究。当前，NETs抑制剂存在一定的副作用，且靶向NETs的肿瘤治疗存在局限，亟需探索新型NETs靶向药物。其次，与中性粒细胞类似，其他免疫细胞也可生成细胞外陷阱（extracellular traps, ETs），并参与各种感染性和非感染性疾病的发展^[93]^，如嗜酸性粒细胞、巨噬细胞、肥大细胞和嗜碱性粒细胞等^[94]^。然而，这些免疫细胞生成的ETs对TME的具体作用尚不完全清楚。综上，今后仍需进一步探索NETs在肿瘤发生发展中的作用，以期为肿瘤靶向治疗提供更加精准的治疗策略。
